# Giant cell tumor of the clavicle: report of a case in a rare location with consideration of surgical method

**DOI:** 10.1186/s12891-015-0604-4

**Published:** 2015-06-12

**Authors:** Satoshi Nagano, Toru Tsuchimochi, Masahiro Yokouchi, Takao Setoguchi, Hiromi Sasaki, Hirofumi Shimada, Shunsuke Nakamura, Yasuhiro Ishidou, Takuya Yamamoto, Setsuro Komiya

**Affiliations:** Department of Orthopaedic Surgery Graduate School of Medical and Dental Sciences, Kagoshima University, 8-35-1 Sakuragaoka, Kagoshima, 890-8520 Japan; The Near-Future Locomotor Organ Medicine Creation Course (Kusunoki Kai), Kagoshima University, 8-35-1 Sakuragaoka, Kagoshima, 890-8520 Japan; Department of Medical Joint Materials, Graduate School of Medical and Dental Sciences, Kagoshima University, 8-35-1 Sakuragaoka, Kagoshima, 890-8520 Japan

**Keywords:** Giant cell tumor, Claviculectomy, Pathology, Shoulder function

## Abstract

**Background:**

Most bone tumors that occur in the clavicle are malignant. A few giant cell tumors (GCTs) of the clavicle have been reported; however, the most appropriate operative method for this tumor has never been discussed.

**Case presentation:**

A 54-year-old man noticed enlargement of the proximal aspect of the right clavicle. A plain X-ray revealed lytic change and ballooning of the proximal end of the right clavicle. The tumor was isointense on T1-weighted magnetic resonance images and showed a mixture of low- and high-intensity areas on T2-weighted images without extension to the surrounding soft tissues. Bone scintigraphy showed strong accumulation (normal/tumor ratio, 2.31), and positron emission tomography revealed strong uptake of fluorine-18-2-fluoro-2-deoxy-d-glucose (SUVmax, 6.0) in the proximal part of the right clavicle. Because we could not completely exclude malignancy, an open biopsy was performed. Pathologically, the tumor comprised mononuclear stromal cells and multinuclear giant cells, resulting in a diagnosis of a GCT of the bone. Although curettage may be considered for such lesions (Campanacci grade II), we chose resection to minimize the chance of recurrence. The tumor was resected en-bloc with the proximal half of the clavicle. No postoperative shoulder disproportion was observed, and full range of motion of the right shoulder was maintained. The patient was satisfied with the surgical outcome (Musculoskeletal Tumor Society score of 96 %). He returned to his original job as a land and house investigator without any signs of recurrence for 1 year after surgery.

**Conclusions:**

Although GCT of the bone rarely occurs in the clavicle, the typical X-ray findings demonstrated in the present case are helpful for a correct diagnosis. Although en-bloc resection without reconstruction is appropriate for GCTs in expendable bones, there has been much discussion about shoulder function after total claviculectomy. Considering the importance of the function of the clavicle, which is to support the scapula through the acromioclavicular joint, we preserved the muscle attachments of the deltoid, trapezius, and pectoralis major. Because both the oncological and functional outcomes were satisfactory, we recommend preservation of as much of the clavicle as possible in patients with clavicular bone tumors.

## Background

Giant cell tumors (GCTs) are aggressive bone tumors comprising osteoclast-like multinuclear cells and hyperplastic mononuclear interstitial cells. In the latest classification of bone tumors by the World Health Organization, GCTs are classified as “intermediate locally aggressive, rarely metastasizing” tumors [1]. Because GCTs show clinically “uncertain behavior” and have a relatively high recurrence rate, the surgical method should be carefully chosen based on the radiographic classification proposed by Campanacci et al. [2]. Sites often affected by GCTs are the distal femur, proximal tibia, and distal radius; GCTs rarely occur in the clavicle [3]. Errani et al. [4] found no GCTs arising in the clavicula among 349 GCTs of bone. However, the national bone tumor registry in Japan reported two cases of GCTs in the clavicula (1.1 %) from 2006 to 2012 [5]. Although bone tumors rarely occur in the clavicle, a high proportion of those that develop at this site are malignant [6, 7]. Therefore, establishing a list of preoperative differential diagnoses of bone tumors involving the clavicles is often difficult. Because the clavicle is a non-weight-bearing bone and is functionally expendable, the optimal surgical resection method for GCTs in this area is controversial. We herein present a case of a GCT in the proximal clavicle. Biopsy was performed to reach a pathological diagnosis after performance of imaging studies, including radionuclide scanning. Functional evaluation after proximal partial claviculectomy demonstrated satisfactory results.

This case has been reported in accordance with the Helsinki Declaration. This retrospective case report is an exemption by the ethics committee of Kagoshima University.

## Case presentation

**Fig. 1:**
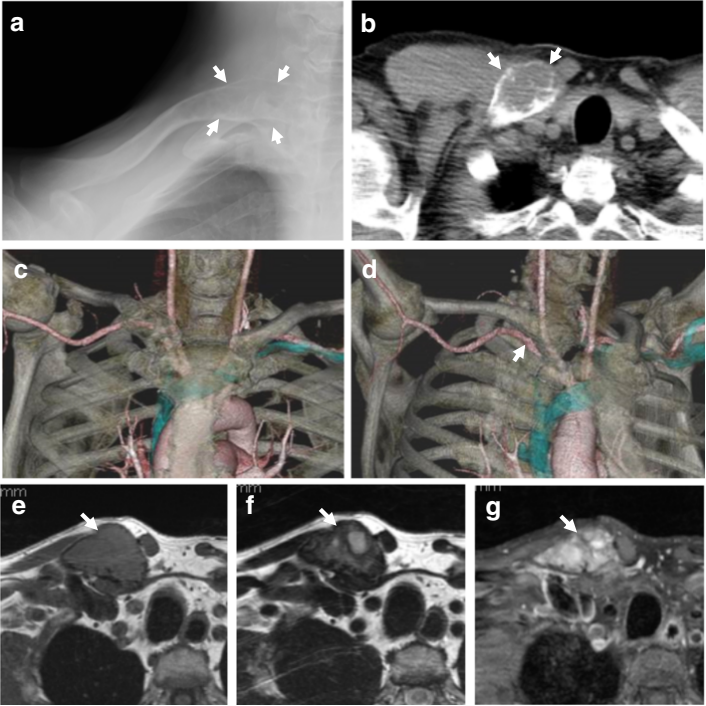
Plain X-ray, computed tomography, and magnetic resonance imaging of the right clavicle. (**a**) Plain X-ray showed lytic change and ballooning of the proximal end of the right clavicle. (**b**) Computed tomography (CT) demonstrated an expanded medullary cavity and thinning of the cortex without periosteal reaction. (**c, d**) 3D-CT angiography images demonstrated proximity of the subclavian artery (*arrow*) and the tumor. The tumor showed (**e**) isointensity on T1-weighted images and (**f**) a mixture of low- and high-intensity areas on T2-weighted images. (**g**) The tumor tissue was significantly enhanced by gadolinium; however, the tumor did not extend to the surrounding soft tissues

**Fig. 2:**
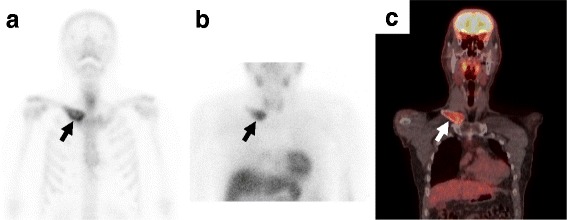
Radionuclear medicine. (**a**) Bone scintigraphy showed marked uptake in the proximal clavicle, and (**b**) thallium-201 scintigraphy showed strong accumulation (normal/tumor ratio, 2.31), suggesting an abundant blood supply to the tumor. (**c**) Positron-emission tomography demonstrated strong accumulation of fluorine-18-2-fluoro-2-deoxy-d-glucose (SUVmax, 6.0) in the proximal part of the right clavicle, but there were no primary tumors or metastases in other body sites

A 54-year-old man noticed enlargement of the proximal aspect of the right clavicle. He made an appointment to undergo positron-emission tomography (PET)-based cancer screening 1 month later, which revealed a lesion with abnormal accumulation in the right clavicle. He was referred to our department for further examination. Plain X-ray revealed lytic change and ballooning of the proximal end of the right clavicle (Fig. 1a). Computed tomography (CT) showed an expanded medullary cavity and thinning of the cortex without periosteal reaction (Fig. 1b). No lung metastasis was demonstrated by thin-slice chest CT. The tumor was isointense on T1-weighted magnetic resonance images and showed a mixture of low- and high-intensity areas on T2-weighted images. However, the tumor did not extend to the surrounding soft tissues (Fig. 1c–e). Bone scintigraphy showed uptake of 99mTc-methylene diphosphonate in the proximal clavicle (Fig. 2a), and thallium-201 scintigraphy showed strong accumulation (normal/tumor ratio, 2.31), suggesting an abundant blood supply to the tumor (Fig. 2b). PET revealed strong accumulation of fluorine-18-2-fluoro-2-deoxy-d-glucose (SUVmax, 6.0) in the proximal part of the right clavicle, but no other primary cancer or metastases were demonstrated in other sites of the body (Fig. 2c). All hematological tumor markers (CA19-9, CEA, AFP, NSE, IL-2R, urinary Bence-Jones protein, and serum M-protein) were negative.

**Fig. 3:**
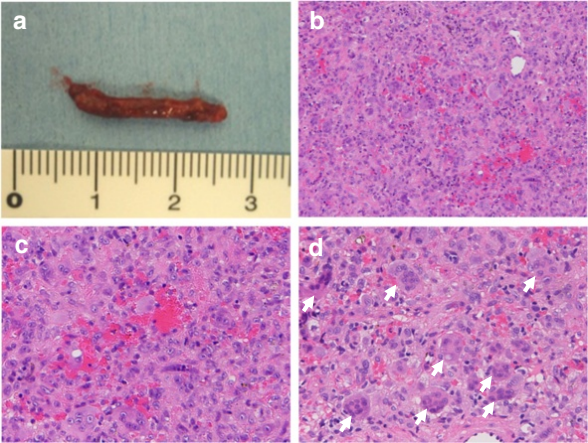
Macroscopic and microscopic biopsy findings. (**a**) Fragile, yellowish-brown tumor tissue was obtained by the biopsy. (**b**) Pathologically, the tumor comprised mononuclear stromal cells and multinuclear giant cells (original magnification, ×100). (**c**) The stromal cells showed oval nuclei with fine, uniform chromatin, and nucleoli were frequently found (original magnification, ×200). Many multinucleated giant cells resembling osteoclasts were surrounded by mononuclear cells (*arrows*)

Based on the characteristic roentgenographic and CT imaging findings with ballooning of the affected bone, the primary differential diagnosis was a GCT. However, we could not completely exclude malignancy because of the affected site, patient age, and degree of accumulation on PET. We thus performed an open biopsy of the tumor. Preoperative angiography was performed to prevent dissemination due to massive perioperative bleeding; however, large nutrient vessels requiring embolization were not demonstrated. Fragile, yellowish-brown tumor tissue was obtained by the biopsy (Fig. 3a). Pathologically, the tumor comprised mononuclear stromal cells and multinuclear giant cells (Fig. 3b). The stromal cells showed oval nuclei with fine, uniform chromatin, and nucleoli were frequently found (Fig. 3c). Many multinucleated giant cells resembling osteoclasts were surrounded by mononuclear cells (Fig. 3c). The case was pathologically diagnosed as a GCT of bone, and surgical treatment was planned.

**Fig. 4:**
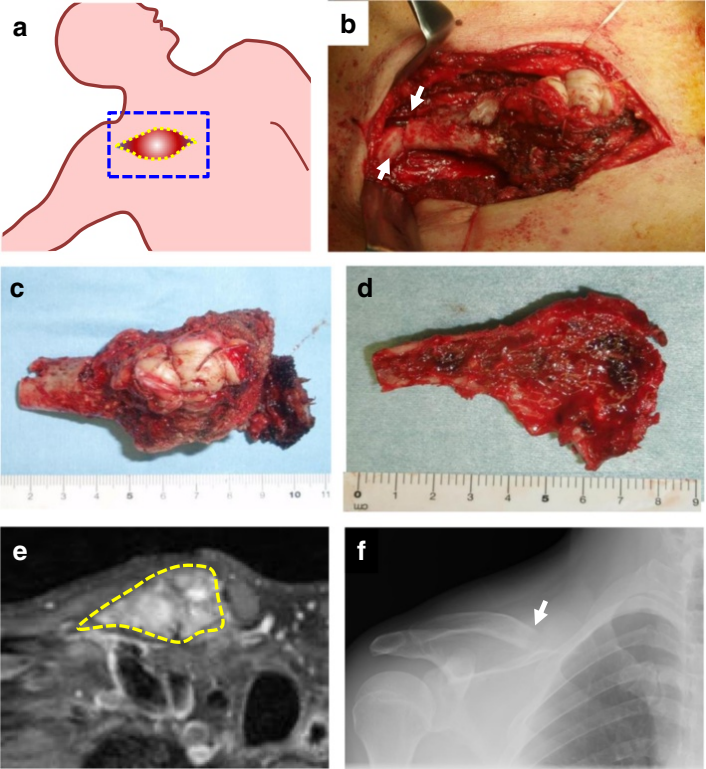
Intraoperative findings. (**a**) In the supine position with the right arm abducted, the right clavicle was carefully exposed without damaging the tumor capsule. (**b**) An osteotomy was made 8 cm from the proximal edge of the clavicle (*arrows*). (**c**) Disarticulation of the sternoclavicular joint was then performed. (**d**) The tumor, resected en-bloc with half of the clavicle, had a thin, fragile cortex, but no extension to the soft tissue. (**e**) The residual clavicular length was 7.5 cm on postoperative roentgenography. (**f**) No displacement of the resection edge of the clavicle was observed

Because this was determined to be a grade II tumor (cortical erosion, deformity, and expansion of bone) according to the Campanacci classification [2], curettage was considered initially. However, we chose resection to minimize the chance of recurrence because adhesion between the clavicle and major vessels was anticipated, which would make reoperation very difficult. Additionally, the clavicle does not necessarily require reconstruction after resection, and this patient was not engaged in physical work. An osteotomy was performed 8 cm from the proximal edge of the clavicle (Fig. 4b), and the sternoclavicular joint was then disarticulated (Fig. 4c). The tumor was resected en-bloc with half of the clavicle. It contained a thin, fragile cortex, but extension to the surrounding soft tissue was absent (Fig. 4d). The residual clavicular length was 7.5 cm on postoperative roentgenography (Fig. 4e), and no displacement of the resection edge of the clavicle was observed (Fig. 4f).

**Fig. 5:**
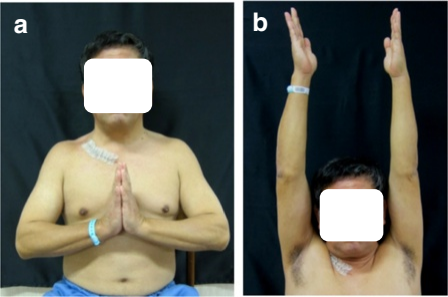
Postoperative appearance and range of motion. (**a**) Disproportion of the shoulder was not observed. (**b**) The range of motion of the right shoulder was normal

No shoulder disproportion was observed postoperatively (Fig. 5a). The range of motion of the right shoulder was normal (Fig. 5b), and the Japanese Orthopaedic Association shoulder score (JOA score) [8] was 99 points. The patient was satisfied with the surgical outcome, and the Musculoskeletal Tumor Society score [9] was 96 %. He returned to his original job as a land and house investigator without any signs of recurrence for 1 year after surgery.

## Conclusions

Dahlin et al. [7] reported that in their study, more than 90 % of bone tumors that occurred in the clavicle were malignant. Other authors have reported high malignancy rates of 51 to 66 % [10, 11], suggesting that physicians should consider the presence of malignancy when a bone tumor is suspected in the clavicle. In a recent large-scale report by Ren et al. [6], the benign/malignant ratio was 1.34 among 206 clavicle-origin bone tumors. In the current case, characteristic roentgen imaging findings were suggestive of a GCT; however, the possibility of malignancy could not be completely excluded because of the imaging findings, including PET, and the rare site of origin. It is known that malignant bone tumors show a high SUVmax in PET. Aoki et al. [12] reported an average SUVmax of 2.2 ± 1.5 and 4.3 ± 3.2 in benign and malignant bone tumors, respectively. GCT of bone, a relatively aggressive bone tumor, shows a high SUV. The average SUV of GCT (4.6 ± 1.1) is reportedly not significantly different from that of osteosarcoma (3.1 ± 1.0) [12]. Preoperative chemotherapy should be considered for malignant bone tumors, including osteosarcoma, Ewing’s sarcoma, and plasmacytoma, and we believe that biopsy should be considered in cases characterized by aggressive behavior. Although the present patient first underwent PET/CT examination by his own choice, this is not a recommended diagnostic procedure for bone tumors in the clavicula. As Rossi et al. [13] described in their recent review of six cases of clavicular bone tumors, plain X-rays, MRI, and total body CT scans are crucial for the diagnosis and staging of clavicular tumors.

A principal surgical treatment method for GCTs, which are benign bone tumors, is curettage. However, the relatively high local recurrence rate (>20 %) is a problem [3, 14, 15]. Various adjuvant treatments, such as liquid nitrogen, ethanol, phenol, and hydrogen peroxide, have been used to reduce the recurrence rate [3, 15]. However, recurrence cannot be completely suppressed, even with the use of adjuvant therapy. Rather, complete removal of the tumor tissue is important. It should also be kept in mind that GCT is associated with a risk of lung metastasis (2 %) [3, 15], and this risk may increase with local recurrence [1]. Most surgeons, including us, mainly perform extensive curettage using a high-speed burr or argon beam coagulator followed by polymethyl methacrylate (PMMA) cementing. The advantages of PMMA cementing are reconstruction of the defect, which allows for immediate weight-bearing, and the ease of identifying recurrence because of the clear border between the PMMA and host bone [3, 15]. In contrast, en-bloc resection should be considered in grade III cases characterized by cortical bone destruction and a soft tissue mass. However, subsequent reconstruction, which sometimes requires a bulky tumor prosthesis, is often problematic [14]. In contrast, en-bloc resection without any reconstruction is performed for GCTs in expendable bones such as the distal ulna, proximal fibula, or iliac wing. Because there are only a few reports of clavicular GCTs [16–19], the optimal surgical method has not reached consensus. However, it seems that claviculectomy, either partial or total, might be a good option for clavicular GCTs. Although partial claviculectomy was chosen in the present case, extensive curettage and PMMA cementing may be considered for younger patients with higher physical activity.

Whether a clavicular bone tumor is malignant or benign is key to selecting the most appropriate surgical method. Obviously, total claviculectomy will be performed for malignant bone tumors [11, 20]. However, postoperative functional loss and risk of recurrence associated with resection should be taken into consideration when selecting the operative method for benign tumors, whether GCTs or other bone tumors. There has been much discussion about shoulder function after total clavicle resection. Krishnan et al. [21] reported that the postoperative function of the affected limb was normal and that only mild pain was present after total claviculectomy. In contrast, Rockwood and Wirth [22] reported unsatisfactory outcomes in most cases (85 %) because of pain, loss of muscle strength, and dropping of the shoulder with or without neurovascular compression or shoulder joint instability; they therefore recommended that surgeons preserve as much of the clavicle as possible. An important function of the clavicle is support of the scapula through the acromioclavicular joint. Muscles such as the deltoid, trapezius, and pectoralis major also attach in this region and serve as a site of action. We resected the proximal clavicle with a margin of 2 cm from the tumor edge, preserving 7.5 cm of the distal clavicle, and were able to maintain a portion of its normal function as described above. Therefore, surgeons should consider how much of the distal part of the clavicle they can preserve to avoid its proximal displacement, which may induce pain or cosmetic issues.

In conclusion, GCT should be considered when the typical X-ray appearance is observed in a patient with an aggressive clavicular bone tumor. If partial resection of the clavicle is necessary, we recommend preservation of as much of the clavicle as possible because no asymmetry, pain, or shoulder imbalance occurred in the present case.

### Consent

Written informed consent was obtained from the patient for publication of this Case report and any accompanying images. A copy of the written consent is available for review by the Series Editor of this journal.

## Abbreviations

GCT: Giant cell tumor
PET: Positron emission tomography
SUV: Standard uptake value
